# Natural Occurrence of *Alternaria* Toxins in Agricultural Products and Processed Foods Marketed in South Korea by LC–MS/MS

**DOI:** 10.3390/toxins14120824

**Published:** 2022-11-24

**Authors:** So Young Woo, Sang Yoo Lee, Tae Kyun Jeong, Su Mi Park, Joong Hyuck Auh, Han-Seung Shin, Hyang Sook Chun

**Affiliations:** 1 School of Food Science and Technology, Chung-Ang University, Anseong 17546, Republic of Korea; 2 Department of Food Science and Biotechnology, Dongguk University-Seoul, Goyang-si 10326, Republic of Korea

**Keywords:** *Alternaria* toxins, method validation, occurrence, food products, South Korea

## Abstract

*Alternaria* mycotoxins including alternariol (AOH), alternariol monomethyl ether (AME), altenuene (ALT), altertoxin-I (ATX-I), tentoxin (TEN), and tenuazonic acid (TeA), are ubiquitous contaminants in agricultural products. A method for the simultaneous determination of these six toxins by ultrahigh performance liquid chromatography–tandem mass spectrometry (LC–MS/MS) with solid phase extraction (SPE) was validated in rice, sesame, tomato, and apple juice matrices. The performance of the method was evaluated in terms of linearity (*R*^2^ > 0.999), the limit of detection (0.04–1.67 μg/kg), the limit of quantification (0.12–5.06 μg/kg), recovery (80.0–114.7%), and precision (<17.7%). The validated method was applied to monitor 152 marketed food samples in South Korea, as well as to investigate the co-occurrence and correlation between *Alternaria* toxins. The mean occurrence levels were 2.77 μg/kg for AOH, 4.36 μg/kg for AME, 0.14 μg/kg for ALT, 0.11 μg/kg for ATX-I, 0.43 μg/kg for TEN, and 104.56 μg/kg for TeA. Mean and extreme (95th percentile) daily dietary exposures of South Koreans to *Alternaria* toxins were estimated to be 22.93 ng/kg b.w./day and 86.07 ng/kg b.w./day, respectively.

## 1. Introduction

*Alternaria* species, so-called black molds, are common fungi of saprophytic origin with pathogenic effects on plants, which cause economic losses and post-harvest spoilage in many crops worldwide. *Alternaria* spp. can produce more than 70 different secondary metabolites (*Alternaria* toxins, ATs) [1]. The *Alternaria alternata* fungus is a major producer of ATs, including dibenzo-α-pyrones (lternariol, (AOH), alternariol monomethyl ether (AME), and altenuene (ALT)), perylene quinones (altertoxin (ATX) I, II, III, and stemphyltoxin III (STTX III)), a cyclic tetrapeptide (tentoxin, (TEN)), a tetramic acid (tenuazonic acid, (TeA)), and *Alternaria alternata* f. sp. *lycopersici* (AAL) toxins [2]. Among these toxins, AOH, AME, ALT, ATX-I, TEN, and TeA, are the main contaminants in agricultural products and have high toxicological significance [3,4]. Previous studies have reported that both AOH and AME are genotoxic and can cause DNA damage in mammalian cells; however, studies of their in vivo toxicity are still limited [5,6,7,8]. ATXs exhibited higher mutagenic activity than AOH and AME in a *Salmonella* Ames test conducted by Schrader et al. (2001) [9]. In contrast, ALT and TeA induce acute toxicity in mice, rats, and chickens, without genotoxicity [10,11].

The determination of ATs has been conducted through a wide variety of methods including enzyme-linked immunosorbent assay (ELISA), thin layer chromatography (TLC), liquid chromatography (LC), and gas chromatography (GC), among others. The GC- mass spectrometry (GC-MS) method showed high sensitivity with a limit of detection of 1 μg/kg for AOH and AME in food matrix [12]. However, most of the ATs are non-volatile compounds, and therefore derivatization is required for GC-MS analysis. Recent research on the determination of ATs has been mainly carried out by liquid chromatography–tandem mass spectrometry (LC–MS), due to its high selectivity and simultaneous determination capabilities [13]. However, the LC–MS method has the disadvantage that the signal can be enhanced or suppressed by the food matrix; hence, it is important to develop an appropriate sample preparation method considering sample dilution, clean-up, and matrix-matched calibration [14]. The solid phase extraction (SPE) method is the most commonly used clean-up technique for liquid extracts from a food matrix and is known to improve the selectivity and performance of the analysis [13]. A few studies on the LC–MS-based analysis of ATs in food with SPE clean-up are available in the literature [15,16,17,18,19]. 

The natural occurrence of ATs in various foods, including cereal grains, legumes, oil seeds, edible oils, vegetables, fruits, and their processed products, has been constantly reported [18,20,21,22,23]. Half of recent studies also reported the occurrence of ATs in fruits and vegetable products, including approximately 40% of the studies on cereal grains and their products, and only ~10% of studies on other food commodities [24]. According to data collected by the European Food Safety Authority (EFSA) in 2016, 20% of the food samples were contaminated with TeA, with the frequency of contamination in samples increasing in the order cereal grains < tomato-based products < cereal grain-based foods for infants/young children [3]. Although many studies have investigated AT contamination in marketed agricultural products, very few studies were performed in South Korea.

Therefore, in this work, we validated an LC–MS/MS-based analytical method for the simultaneous determination of six ATs (AOH, AME, ALT, ATX-I, TEN, and TeA) to monitor the natural occurrence of ATs in agricultural products and processed foods marketed in South Korea. The co-occurrence of ATs and correlation between ATs in various food products were investigated. Additionally, daily dietary exposure of South Koreans to ATs was estimated.

## 2. Results and Discussion

### 2.1. Method Validation

#### 2.1.1. Selectivity, Linearity, Limit of Detection, and Limit of Quantification

**Figure 1:**
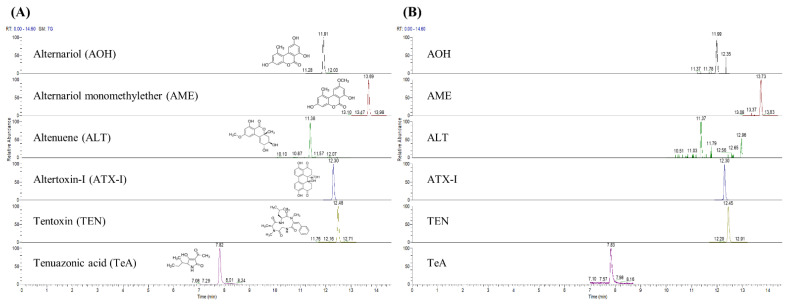
Chromatograms of *Alternaria* toxins. (**A**) The concentrations of the standards were as follows: 10 μg/kg for AOH, AME, ALT, ATX-I; 50 μg/kg for TEN; 100 μg/kg for TeA. (**B**) Chromatograms of contaminated sesame sample.

Among agricultural products and processed foods, rice, sesame, tomato, and apple juice were selected as representative matrices of cereal grains, nuts and seeds, fruits, and processed products, respectively. Moreover, six *Alternaria* toxins were successfully isolated through chromatographic separation (Figure 1). The linearity of the method was evaluated using the coefficient of determination (*R*^2^) of the matrix-matched external calibration curve. The six-point calibration curves in the ranges of 1–50 μg/kg (for AOH, AME, ALT, and ATX-I), 5–250 μg/kg (TEN), and 10–500 μg/kg (TeA) showed excellent linearities (*R*^2^ > 0.999) for all four food matrices. The calculated limit of detection (LOD) and the limit of quantification (LOQ) were 0.12–0.67 and 0.35–2.04 μg/kg for rice, 0.05–1.44 and 0.14–4.35 μg/kg for sesame seed, 0.09–1.67 and 0.26–5.06 μg/kg for tomato, as well as 0.04–0.58 and 0.12–1.76 μg/kg for apple juice, respectively (Table 1).

#### 2.1.2. Recovery and Precision

The *Alternaria* toxins were classified into three different groups based on the similarity of their reported occurrence levels. For validation, the rice, sesame, tomato, and apple juice samples were spiked with 5, 10, and 20 μg/kg of AOH, AME, ALT, and ATX-I, 25, 50, 100 μg/kg of TEN, and 50, 100, 200 μg/kg of TeA. Each value was rounded to the first decimal place. The intra- and inter-day validation results are listed in Table 2.

The mean inter- and intra-recovery ranges of the six analytes in rice, sesame seed, tomato, and apple juice were 82.1–114.7%, 80.0–94.9%, 82.7–110.7%, and 85.9–114.3% ranges, respectively. The mean relative standard deviation (RSD), an estimate for the precision, of the six analytes ranged from 4.3 to 15.6% in rice, 2.5 to 17.7% in sesame, 3.2 to 15.1% in tomato, and 4.1 to 15.4% in apple juice. All of the estimated recovery and precision values were within an acceptable range according to the AOAC Official Methods of Analysis (2016) Guidelines for Standard Method Performance Requirements. Specifically, the guideline recommend that the recovery values should be within 60–115% and 80–110% for concentrations of 10 and 100 μg/kg, respectively [25]. Moreover, the RSD values recommended by the AOAC for concentrations of 10 and 100 μg/kg are <21% and <15%, respectively.

### 2.2. Occurrence of Alternaria Toxins in Agricultural Products and Processed Foods

**Figure 2:**
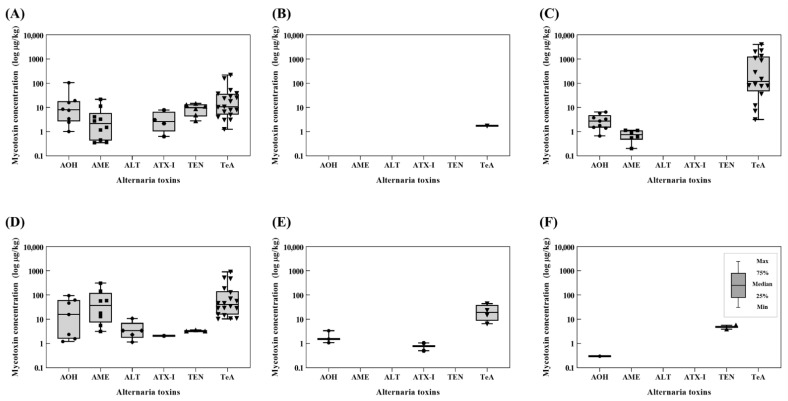
Prevalence of *Alternaria* toxins quantified in different food categories: (**A**) cereal grains, (**B**) pulses, (**C**) seasoning foods, (**D**) nuts and seeds, (**E**) beverages, and (**F**) vegetables and fruits (each dot within the boxplots indicates one positive sample).

A total of 152 marketed food samples, including cereal grains (*n* = 31), pulses (*n* = 15), seasoning foods (*n* = 16), nuts and seeds (*n* = 22), beverages (*n* = 32), vegetables (*n* = 16), and fruits (*n* = 20) were analyzed using the validated method. The obtained contamination levels of *Alternaria* toxins are reported in Figure 2 and Table 3, which include the incidence, mean concentrations, and concentration ranges of the six *Alternaria* toxins in each food product.

**Figure 3:**
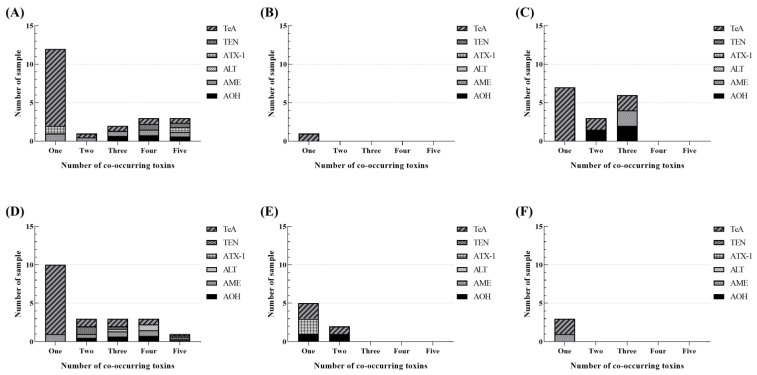
Co-occurrence of *Alternaria* toxins in different food categories: (**A**) cereal grains, (**B**) pulses, (**C**) seasoning foods, (**D**) nuts and seeds, (**E**) beverages, and (**F**) vegetables and fruits.

Among the dibenzo-α-pyrone derivatives, the highest incidence was found for AOH (27/152), followed by AME (25/152) and ALT (5/152). The mean concentration of AOH in all of the tested samples was 2.77 μg/kg, with levels ranging from 0.66 to 105.49 μg/kg. The food product with the highest occurrence of AOH was buckwheat, which was also the food product with the highest incidence (mean = 26.68 μg/kg, maximum = 105.49 μg/kg, incidence = 80%). The mean concentration of AME in all of the tested samples was 4.36 μg/kg, with a range of 0.20 to 310.82 μg/kg, which was slightly wider than that of AOH. The highest concentration of AME was found in black sesame (mean = 106.49 μg/kg, maximum = 310.82 μg/kg, incidence = 80%), and the food product with the highest AOH detection frequency was buckwheat (mean = 6.18 μg/kg, maximum = 21.89 μg/kg, incidence = 100%). ALT was detected in five samples of the nuts and seeds group, whereas none of the tested samples of cereal grains, pulses, seasoning foods, beverages, vegetables, and fruits was contaminated with ALT. The mean occurrence level of ALT was 0.14 μg/kg, with levels ranging from 1.13 to 10.71 μg/kg. The highest level of ALT was determined in black sesame (mean = 3.95 μg/kg, maximum = 10.71 μg/kg, incidence = 80%). Interestingly, all of the detected ALT in the examined food samples co-occurred with at least two other *Alternaria* toxins (Figure 3).

ATX-I, a perylene quinone derivative, was detected in four samples of cereal grains, one sample of nuts and seeds, and two samples of beverages. ATX-I was not found in any of the examined samples of pulses, seasoning foods, vegetables, and fruits. The mean concentration of ATX-I in all monitored foods was 0.11 μg/kg, and its levels ranged from 0.50 to 7.81 μg/kg; this was the lowest contamination level among the six *Alternaria* toxins. The highest contamination level and incidence of ATX were found in buckwheat (mean = 2.17 μg/kg, maximum = 7.81 μg/kg, incidence = 40%).

TEN, a cyclic tetrapeptide, was found in all monitored samples at levels ranging from 2.77 to 14.64 μg/kg. The average TEN contamination level found in the 152 tested samples was 0.43 μg/kg, which was similar to that of ALT and ATX-I. Only nine samples were positive for TEN, whose highest concentration and incidence were observed in sorghum (mean = 3.48 μg/kg, maximum = 14.64 μg/kg, incidence = 40%) and buckwheat (mean = 5.75 μg/kg, maximum = 13.00 μg/kg, incidence = 60%). All ALT-positive samples were co-contaminated with other *Alternaria* toxins (Figure 3). The levels of ATX-I, ALT, and TEN in marketed foods from South Korea were similar to or lower than those recently reported by Xing et al. (2021), Fan et al. (2022), and Zhao et al. (2022) [21,26,27].

TeA, which is classified as a tetramic acid, was a major contaminant in this study. Among the 152 monitored samples, 39.5% (60/152) were contaminated with TeA at concentrations ranging from 1.26 to 4028.18 μg/kg, with a mean value of 104.56 μg/kg. TeA was detected in all of the 16 examined seasoning foods, and hot pepper powder was a major contributor to the TeA contamination (mean = 1845.95 μg/kg, maximum = 4028.18 μg/kg, incidence = 100%). Furthermore, the five food products with the highest concentration of TeA were also all red pepper powder products (1104.98–4028.18 μg/kg). This represents a lower occurrence level than those previously reported by Gambacor-ta et al. (2019) [28] (mean concentration in red chili = 27,255.5 μg/kg) and Mujahid et al. (2020) [29] (maximum concentration in chili items = 20,478 μg/kg). The incidence of TeA was high in the following order: black sesame (mean = 335.66 μg/kg, maximum = 911.63 μg/kg, incidence = 100%), tomato puree (mean = 211.07 μg/kg, maximum = 882.22 μg/kg, incidence = 100%), perilla seed (mean = 138.64 μg/kg, maximum = 476.19 μg/kg, incidence = 100%), and tomato ketchup (mean = 73.26 μg/kg, maximum = 91.10 μg/kg, incidence = 100%). Among the seasoning foods investigated, the contamination levels of TeA in tomato products (ketchup and puree) were similar to or lower than those reported in previous studies [10,22,30,31].

### 2.3. Correlation between Naturally Occurring Alternaria Toxins

**Figure 4:**
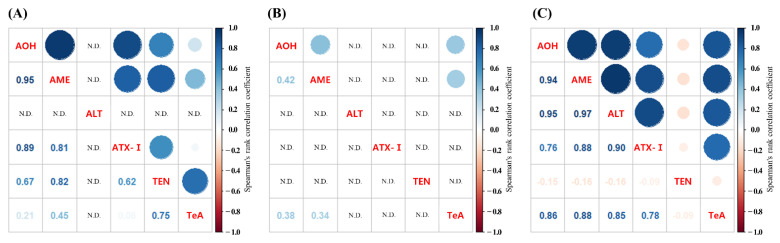
Correlation matrix of the concentration of *Alternaria* toxins in the most frequently contaminated food categories: (**A**) cereal grains, (**B**) seasoning foods, (**C**) nuts and seeds.

The correlations between naturally co-occurring *Alternaria* toxins were investigated in cereal grain, seasoning food, and nuts and seeds, representing the three most frequently contaminated food categories (Figure 4). The correlation between *Alternaria* toxins was visualized using the ‘corrplot’ R package (version 4.1.2).

ALT was not detected in any of the cereal grain samples (*n* = 31), and a positive correlation was observed between the contamination levels of all detected *Alternaria* toxins. The Spearman’s rank correlation coefficients (*r*_s_) were 0.95 (AOH and AME), 0.89 (AOH and ATX-I), 0.82 (AME and TEN), 0.81 (AME and ATX-I), 0.75 (TEN and TeA), 0.67 (AOH and TEN), 0.62 (ATX-I and TEN), 0.45 (AME and TeA), 0.21 (AOH and TeA), and 0.06 (ATX- I and TeA). These results indicated that the wheat and wheat-based products examined herein showed a similar trend to those reported by Zhao et al. (2015) and Xu et al. (2016) [16,17].

Only three types of *Alternaria* toxins, AOH, AME, and TeA, were detected in seasoning foods. In this food group, TeA co-occurred with AOH and/or AME in 56.3% of the samples, and a particularly high contamination level of TeA was observed. However, only a weak positive correlation was observed between the detected toxins (*r*_s_ = 0.42 for AOH and AME, 0.38 for AOH and TeA, and 0.34 for AME and TeA).

All six *Alternaria* toxins were detected in the nuts and seeds group. A high positive correlation was observed between the dibenzo-α-pyrone derivatives (*r*_s_ ≥ 0.94). Moreover, a positive correlation was observed between all *Alternaria* toxins except for TEN, and the *r*_s_ value between the toxins showing a positive correlation was higher than 0.76. Interestingly, TEN showed a weak negative correlation with all other *Alternaria* toxins only in the nuts and seeds group. To the best of our knowledge, our study is the first to demonstrate that the contamination levels of *Alternaria* toxins may show a negative correlation depending on the food category. Particularly, the *r*_s_ values were −0.16 (AME and TEN, ALT and TEN), −0.15 (AOH and TEN), and −0.09 (ATX-I and TEN, TeA and TEN).

### 2.4. Estimation of Daily Dietary Exposure to Alternaria Toxins

The estimated daily exposure of each ATs from marketed food in the South Korean population is summarized in Table 4. For the mean exposure scenario, the average daily exposure levels were 0.0118 (LB)–0.0506 ng/kg b.w./day (UB) for AOH, 0.0101 (LB)–0.0378 ng/kg b.w./day (UB) for AME, 0.0001 (LB)–0.0364 ng/kg b.w./day (UB) for ALT, 0.0022 (LB)–0.0381 ng/kg b.w./day (UB) for ATX-I, 0.0039 (LB)–0.1744 ng/kg b.w./day (UB) for TEN, and 3.7094 (LB)–3.8499 ng/kg b.w./day (UB) for TeA in all food samples. Seasoning foods were the main contributors for dietary exposure to AOH and TeA. Nuts and seeds were the major contributors for dietary exposure to AME and ALT. Beverages and cereal grains were the highest contributors for daily dietary exposure to ATX-I and TEN, respectively.

For the extreme exposure (95th percentile) scenario, the average daily exposure levels were 0.0935 (LB)–0.2255 ng/kg b.w./day (UB) for AOH, 0.0733 (LB)–0.1572 ng/kg b.w./day (UB) for AME, 0.0003 (LB)–0.1534 ng/kg b.w./day (UB) for ALT, 0.0072 (LB)–0.1889 ng/kg b.w./day (UB) for ATX-I, 0.0591 (LB)–0.8437 ng/kg b.w./day (UB) for TEN, and 14.3004 (LB)–15.0344 ng/kg b.w./day (UB) for TeA in all food samples. Cereal grains were the most important contributors for dietary exposure to AOH, AME, ATX-I, and TEN estimated by extreme food intake scenario due to their high consumption. Nuts and seeds and seasoning foods were the highest contributors for daily dietary exposure to ALT and TeA, respectively.

The estimated exposure levels of ATs in this study were much lower than the previously reported studies based on mean food consumption of Zhao et al. (2015) (3.56 ng/kg b.w./day (LB) for AOH) and EFSA (2016) (0.4–1.9 ng/kg b.w./day (LB) for AOH, 0.7–3.4 ng/kg b.w./day (LB) for AOH, 0.4–1.6 ng/kg b.w./day (LB) for TEN, and 37–100 ng/kg b.w./day (LB) for TeA) [3,16].

## 3. Conclusions

Our study validated an LC-MS/MS-based method with SPE clean-up for the simultaneous analysis of six ATs in four different food matrices to investigate the natural occurrence of ATs in marketed foods in South Korea. Further, the co-occurrence of six ATs and correlation between different levels of toxin were investigated. Processed tomato products are among the foods that are susceptible to contamination by *Alternaria* toxins. In South Korea, the occurrence of toxins in processed tomato products (ketchup, puree, juice) was similar to those reported in other countries. TeA was the most frequently detected *Alternaria* toxin among all of the tested food categories, and the highest TeA level was found in hot pepper powder. Although the toxin occurrence levels were lower than those reported in previous studies, continuous monitoring is needed for risk management of red pepper powder, which is among the most widely consumed products in South Korea. Additionally, 44% of positive samples were co-contaminated with at least two *Alternaria* toxins. The overall estimated daily dietary exposures of South Koreans to ATs were lower than those reported in previous studies from other countries. Collectively, our results highlight the need for further monitoring and risk assessment of co-occurring *Alternaria* toxins in agricultural and food products.

## 4. Materials and Methods

### 4.1. Sample Collection

A total of 152 marketed agricultural products and processed food samples were purchased from online retailers, supermarkets, and local markets in 2020. The collected samples were classified into as followings: cereal grains (rice, brown rice, wheat flour, barley, sorghum, and buckwheat), pulses (soybean, kidney bean, and lentils), seasoning foods (hot pepper powder, tomato ketchup, and tomato puree), nuts and seeds (sesame, perilla seed, black sesame, and sunflower seed), beverages (apple juice, orange juice, grape juice, tomato juice, black tea, corn silk tea, barley tea, and soybean milk), vegetables (tomato, onion, and Korean cabbage), and fruits (apple, mandarin, grape, and watermelon). The samples were homogenized and stored at −20 °C, followed by equilibration at room temperature prior to analysis.

### 4.2. Chemicals and Reagents

Certified mycotoxin standards were purchased as follows: AOH from *Alternaria* sp. (purity >96%), AME from *Alternaria alternata* (>98%), TeA copper salt from *Alternaria alternata* (>98%), and TEN from *Alternaria tenuis* (>95%) were obtained from Sigma-Aldrich (St. Louis, MO, USA); ALT (>98%) was purchased from ChemFaces (Wuhan, China), and ATX- I (>97%) was obtained from Cayman (Ann Arbor, MI, USA). All mycotoxin standards were prepared at 1 mg/mL in acetonitrile (ACN) and kept at −20 °C. HPLC-grade water, ACN, and methanol (MeOH) from Honeywell Burdick and Jackson (Muskegon, MI, USA) were used for sample preparation. LC/MS-grade water and methanol from Fisher Scientific (Cleveland, OH, USA) were used as the mobile phase. LC/MS-grade ammonium acetate was purchased from Merck (Darmstadt, Germany) and glacial acetic acid (≥99.7%, HPLC grade) for pH adjustment was obtained from Fisher Scientific (Cleveland, OH, USA).

### 4.3. Sample Preparation

After purification of the *Alternaria* toxins from rice, sesame seed, apple juice, and tomato, toxin determination in tomato, wheat, and sunflower seeds was performed by SPE clean-up and HPLC–MS/MS, using the EN 17521-2021 standard method developed by the Slovenian Institute for Standardization (SIST), with some modifications [32]. Briefly, 2 g of sample was extracted with 15 mL of MeOH/water/acetic acid (85:14:1, *v*/*v*/*v*) by shaking for 45 min at 270 rpm, followed by centrifugation of the extract for 10 min at 3100× *g*. The supernatant (7.5 mL) was diluted with an equal volume of 1% acetic acid solution (*v*/*v*). The *Alternaria* toxins were cleaned up using solid-phase extraction with polymer-based hydrophilic-lipophilic balanced (HLB) SPE cartridges (200 mg, 6 mL) purchased from Waters (Milford, MA, USA). The cartridges were conditioned with 7 mL of MeOH and equilibrated with 7 mL of water and 4 mL of 1% acetic acid. After the equilibration solvent completely passed through the cartridges, their bottom was closed and 3 mL of 1% acetic acid was added. A syringe was attached to the cartridge to load the diluted supernatant and allow the cartridge to flow. Then, the cartridge was washed with 7 mL of 2% Tween 20 (*v*/*v*) (Sigma-Aldrich) and thoroughly dried under a vacuum. Another 7 mL of 1% acetic acid solution (*v*/*v*) was added to wash the cartridge, followed by drying thoroughly under a vacuum. After that, 7 mL of MeOH/ethyl acetate (75:25, *v*/*v*) was added to the cartridge to elute the *Alternaria* toxins. The eluate was evaporated under nitrogen gas at 50 °C and reconstituted with 0.4 mL MeOH and 0.6 mL of 5 mM ammonium acetate (pH 8.6). The reconstituted solvent was filtered through a 0.2 μm PTFE syringe filter. The filtrate was centrifuged (13,000× *g*, 10 min) and the supernatant was injected into the LC-MS/MS system.

### 4.4. LC-MS/MS Equipment and Parameters

Detection and quantification were performed using a Thermo Scientific Vanquish HPLC system coupled with a Thermo Scientific Q Exactive Orbitrap spectrometer (Thermo Fisher Scientific, Waltham, MA, USA) with parallel reaction monitoring in negative electrospray ionization (ESI) mode. Analysis was conducted with a Supelco Ascentis^®^ Express C18 column (4.6 mm × 150 mm, 2.7 μm) connected to a guard column (4.6 mm × 50 mm, 2.7 μm). Separation was conducted over a period of 20 min using a flow rate of 0.4 mL/min: 90% solvent A (5 mM ammonium acetate in water, pH 8.6) and 10% solvent B (MeOH) for 1 min to reach equilibrium. Solvent B was first applied using a linear gradient elution system at a concentration increasing from 10% to 100% over 9 min, and then held for 2 min. The concentration of solvent B was then changed to 10% within 0.2 min, and the column was re-equilibrated for 20 min. The column oven temperature was held at 40 °C. The normalized collision energy was 42%, and nitrogen was used as the collision gas. The spray voltage was −2.5 kV. The source heater and capillary temperatures were set at 250 and 320 °C, respectively, whereas the sheath and auxiliary gas flow rates were 40 and 10 L/min, respectively. Data analysis was performed using the Thermo Xcalibur Qual Browser 3.0 software. All analyzed *Alternaria* toxins were ionized in negative mode in the form of (M − H)^−^ ions. The product ions for quantification (identification) of the six toxins were as follows: AOH 257.045 *m*/*z* → 215.035 *m*/*z* (147.044 *m*/*z*), AME 271.061 *m*/*z* → 256.038 *m*/*z* (228.042 *m*/*z*), ALT 291.087 *m*/*z* → 248.069 *m*/*z* (203.034 *m*/*z*), ATX- I 351.087 *m*/*z* → 315.066 *m*/*z* (333.077 *m*/*z*), TEN 413.219 *m*/*z* → 141.066 *m*/*z* (214.074 *m*/*z*), TeA 196.097 *m*/*z* → 138.019 *m*/*z* (178.087 *m*/*z*).

### 4.5. Method Validation

Standard curves for each mycotoxin were evaluated based on the *R*^2^ value of six-point matrix-matched calibration curves, constructed by plotting the peak areas (signal intensities). The LOD and LOQ values were determined using the slope of the calibration curve (*s*) and the standard deviation (*σ*) of the peak area corresponding to the lowest concentration in the calibration curve, according to the following equations: LOD = 3.3 × *σ*/*s* and LOQ = 10 × *σ*/*s*. The recovery was evaluated by fortification experiments according to the following equation: analyzed concentration of spiked samples calculated from matrix-matched standard/spiking concentration × 100 (%). The precision values were calculated as the RSDs of the replicated recovery experiments.

### 4.6. Estimation of Daily Dietary Exposure

Food consumption data and mean body weight were provided by the Korean National Health and Nutrition Examination Survey (KNHANES, 2020) [33]. The food consumption data included the mean and extreme (95th percentile) in the general population. A lower bound (LB) and upper bound (UB) approach were used for the estimation of dietary exposure. “Not detected (N.D.)” samples that quantified below LOD were evaluated by assigning the N.D. sample as a value of zero (lower bound, LB) and a value of LOD for each AT (upper bound, UB). Daily dietary exposure to ATs was calculated using the following formula: Daily dietary exposure (ng/kg b.w./day) = Mycotoxin contamination level in food (μg/kg) × food intake of the population (g/day)/mean body weight of the population (kg).

## Figures and Tables

**Table 1:** Linearity, LOD, and LOQ parameters in each validated food matrix.

Matrix	Chemical Group	Toxin	Slope	Intercept	*R* ^2^	LOD (μg/kg)	LOQ (μg/kg)
Rice	Dibenzo-α-pyrone derivatives	AOH	38,024.7	−4477.4	1.000	0.42	1.26
		AME	557,506.5	525.4	1.000	0.23	0.69
		ALT	21,640.4	−5656.4	1.000	0.22	0.67
	Perylene quinone derivatives	ATX-I	81,060.5	−28,786.2	0.999	0.12	0.35
	Cyclic tetrapeptide	TEN	183,111.4	241,902.1	1.000	0.67	2.04
	Tetramic acid	TeA	20,620.1	−23,613.1	1.000	0.38	1.16
Sesame	Dibenzo-α-pyrone derivatives	AOH	37,644.2	−5566.9	1.000	0.35	1.06
		AME	481,012.8	296,681.0	0.999	0.62	1.87
		ALT	14,857.9	−6783.0	1.000	0.05	0.14
	Perylene quinone derivatives	ATX-I	77,708.8	−14,826.9	1.000	0.15	0.46
	Cyclic tetrapeptide	TEN	165,058.5	145,868.5	1.000	1.13	3.42
	Tetramic acid	TeA	18,454.9	−2897.7	1.000	1.44	4.35
Tomato	Dibenzo-α-pyrone derivatives	AOH	35,967.4	−14,170.1	1.000	0.13	0.40
		AME	651,313.1	−9161.5	1.000	0.09	0.26
		ALT	19,632.1	−6452.2	1.000	0.24	0.72
	Perylene quinone derivatives	ATX-I	85,741.1	−16,991.5	1.000	0.35	1.05
	Cyclic tetrapeptide	TEN	239,237.6	194,783.3	1.000	1.42	4.30
	Tetramic acid	TeA	21,292.1	−36,110.6	1.000	1.67	5.06
Apple juice	Dibenzo-α-pyrone derivatives	AOH	36,592.4	−3876.9	1.000	0.08	0.24
		AME	460,716.3	80,147	1.000	0.22	0.67
		ALT	18,358.9	−3988.7	1.000	0.12	0.36
	Perylene quinone derivatives	ATX-I	92,791.6	156.6	1.000	0.04	0.12
	Cyclic tetrapeptide	TEN	205,537.6	543,369.8	0.999	0.58	1.76
	Tetramic acid	TeA	20,161.3	−21,619.0	1.000	0.55	1.68

**Table 2:** Recovery and RSD values in each validated food matrix.

Matrix	Parameter	Spiking Level (μg/kg) *	Recovery (%) ± RSD%																	
			Dibenzo-α-Pyrone									Perylene Quinone			Cyclic Tetrapeptide			Tetramic Acid		
			AOH			AME			ALT			ATX-I			TEN			TeA		
Rice	Intra-day	I	101.5	±	11.1	95.3	±	8.9	95.6	±	8.9	114.7	±	9.1	101.7	±	9.9	90.4	±	6.5
		II	96.6	±	5.3	89.4	±	5.7	90.8	±	4.9	101.3	±	9.2	94.9	±	5.0	87.8	±	6.4
		III	99.0	±	5.0	93.3	±	10.1	97.3	±	4.3	105.6	±	10.9	98.6	±	7.6	88.0	±	6.4
	Inter-day	I	96.3	±	12.1	87.0	±	9.6	89.6	±	11.9	102.8	±	11.7	88.5	±	11.5	90.1	±	8.6
		II	101.8	±	11.8	87.4	±	8.6	96.1	±	9.8	99.9	±	13.2	98.7	±	11.1	96.9	±	11.1
		III	102.2	±	10.7	82.1	±	9.3	100.3	±	13.2	97.1	±	11.3	97.0	±	12.6	94.4	±	15.6
Sesame	Intra-day	I	80.0	±	6.3	82.2	±	8.0	87.6	±	5.6	84.3	±	4.6	86.6	±	4.6	94.9	±	13.8
		II	80.5	±	5.6	82.0	±	7.6	85.5	±	2.5	82.8	±	3.6	86.9	±	2.8	81.5	±	8.7
		III	86.8	±	8.7	85.4	±	4.8	89.3	±	5.1	86.7	±	2.9	93.0	±	3.3	84.9	±	5.3
	Inter-day	I	85.3	±	9.6	86.1	±	11.9	90.4	±	9.4	85.7	±	7.4	86.5	±	8.5	91.8	±	17.7
		II	82.7	±	5.0	80.2	±	6.9	87.8	±	8.3	83.6	±	4.8	86.1	±	5.6	82.3	±	8.4
		III	89.9	±	8.5	84.8	±	6.0	90.2	±	4.9	88.0	±	2.9	94.2	±	3.6	85.6	±	9.9
Tomato	Intra-day	I	108.5	±	11.0	97.2	±	8.0	95.7	±	8.0	96.5	±	12.0	87.9	±	8.9	88.0	±	7.3
		II	100.0	±	11.7	91.9	±	7.4	95.6	±	11.1	95.7	±	15.1	89.1	±	11.2	84.1	±	11.6
		III	97.4	±	13.7	104.3	±	9.5	105.9	±	7.0	102.5	±	6.6	100.9	±	6.9	90.3	±	8.9
	Inter-day	I	102.2	±	10.6	87.2	±	13.9	101.2	±	9.2	92.2	±	13.8	89.9	±	6.2	85.0	±	3.2
		II	99.4	±	10.6	89.8	±	7.8	92.0	±	6.7	89.6	±	6.9	86.6	±	7.4	82.7	±	7.4
		III	107.1	±	10.1	107.0	±	10.5	110.7	±	6.8	99.2	±	9.4	106.3	±	9.1	87.4	±	4.1
Apple juice	Intra-day	I	96.0	±	11.3	108.6	±	12.9	114.3	±	9.6	108.1	±	9.3	95.5	±	5.6	87.6	±	12.2
		II	92.5	±	15.1	101.1	±	12.4	103.1	±	9.5	101.7	±	9.9	92.4	±	7.5	86.1	±	9.8
		III	104.5	±	8.5	103.7	±	12.1	108.9	±	12.3	101.1	±	12.7	93.3	±	9.1	88.7	±	6.9
	Inter-day	I	88.6	±	15.4	111.0	±	9.7	105.9	±	10.5	93.7	±	10.2	88.0	±	13.0	90.9	±	13.9
		II	85.9	±	4.1	105.5	±	10.3	108.9	±	14.1	97.5	±	8.8	93.8	±	10.9	88.0	±	9.4
		III	100.0	±	7.5	110.4	±	9.2	114.1	±	5.8	106.8	±	8.8	93.1	±	8.1	95.2	±	13.0

* Spiking level I: 5 μg/kg for AOH, AME, and ALT; 25 μg/kg for TEN; 50 μg/kg for TeA. Spiking level II: 10 μg/kg for AOH, AME, and ALT; 50 μg/kg for TEN; 100 μg/kg for TeA. Spiking level III: 20 μg/kg for AOH, AME, and ALT; 100 μg/kg for TEN; 200 μg/kg for TeA.

**Table 3:** Occurrence of *Alternaria* toxins in commercial food commodities.

Category	Food Product	Concentration (μg/kg)																	
		AOH			AME			ALT			ATX-I			TEN			TeA		
		Mean	Incidence ^1^	Range	Mean	Incidence	Range	Mean	Incidence	Range	Mean	Incidence	Range	Mean	Incidence	Range	Mean	Incidence	Range
Cereal grains	Rice	. ^2^	0/6	.	.	0/6	.	.	0/6	.	.	0/6	.	.	0/6	.	3.84	5/6	1.26–10.68
	Brown rice	.	0/5	.	.	0/5	.	.	0/5	.	.	0/5	.	.	0/5	.	5.18	2/5	7.89–18.01
	Wheat flour	1.53	1/5	7.63	0.56	1/5	2.78	.	0/5	.	0.56	2/5	0.63–2.18	1.74	1/5	8.72	32.67	2/5	4.00–159.34
	Barley	.	0/5	.	0.07	1/5	0.35	.	0/5	.	.	0/5	.	.	0/5	.	16.81	3/5	8.04–52.17
	Sorghum	4.53	3/5	1.01–19.17	2.63	3/5	0.36–11.29	.	0/5	.	.	0/5	.	3.48	2/5	2.77–14.64	51.73	3/5	10.38–219.56
	Buckwheat	26.68	4/5	3.41–105.49	6.18	5/5	0.45–21.89	.	0/5	.	2.17	2/5	3.06–7.81	5.75	3/5	4.80–13.00	21.65	4/5	6.73–38.51
	Total	5.28	8/31	1.01–105.49	1.52	10/31	0.35–21.89	.	0/31	.	0.44	4/31	0.63–7.81	1.77	6/31	2.77–14.64	21.39	19/31	1.26–219.56
Pulses	Soybean	.	0/5	.	.	0/5	.	.	0/5	.	.	0/5	.	.	0/5	.	.	0/5	.
	Kidney bean	.	0/5	.	.	0/5	.	.	0/5	.	.	0/5	.	.	0/5	.	0.35	1/5	1.75
	Lentils	.	0/5	.	.	0/5	.	.	0/5	.	.	0/5	.	.	0/5	.	.	0/5	.
	Total	.	0/15	.	.	0/15	.	.	0/15	.	.	0/15	.	.	0/15	.	0.12	1/15	1.75
Seasoning foods	Hot pepper powder	2.08	4/6	0.66–5.76	0.41	4/6	0.20–1.12	.	0/6	.	.	0/6	.	.	0/6	.	1845.94	6/6	288.47–4028.18
	Tomato ketchup	0.94	3/5	1.41–1.80	0.41	2/5	0.88–1.14	.	0/5	.	.	0/5	.	.	0/5	.	73.26	5/5	36.32–91.10
	Tomato puree	2.05	2/5	3.76–6.48	.	0/5	.	.	0/5	.	.	0/5	.	.	0/5	.	211.07	5/5	3.17–882.22
	Total	1.71	9/16	0.66–6.48	0.28	6/16	0.20–1.14	.	0/16	.	.	0/16	.	.	0/16	.	781.08	16/16	3.17–4028.18
Nuts and Seeds	Sesame	0.17	1/7	1.41–3.30	11.21	4/7	3.12–56.91	0.16	1/7	1.13	.	0/7	.	.	0/7	.	15.53	5/7	10.74–56.49
	Perilla seed	0.31	1/5	1.56–1.56	.	0/5	.	.	0/5	.	.	0/5	.	0.64	1/5	3.21	138.64	5/5	28.91–476.19
	Black sesame	43.85	4/5	15.67–94.97	106.49	4/5	17.82–310.82	3.95	4/5	2.29–10.71	0.41	1/5	2.04	.	0/5	.	335.66	5/5	10.31–911.63
	Sunflower seed	0.47	1/5	2.34	.	0/5	.	.	0/5	.	.	0/5	.	1.36	2/5	3.20–3.58	30.29	3/5	34.34–70.87
	Total	10.20	7/22	1.41–94.97	27.77	8/22	3.12–310.82	0.95	5/22	1.13–10.71	0.09	1/22	2.04	0.45	3/22	3.20–3.58	119.62	18/22	10.31–911.63
Beverages	Apple juice	.	0/5	.	.	0/5	.	.	0/5	.	.	0/5	.	.	0/5	.	.	0/5	.
	Orange juice	.	0/5	.	.	0/5	.	.	0/5	.	.	0/5	.	.	0/5	.	4.31	2/5	6.47–15.10
	Grape juice	.	0/5	.	.	0/5	.	.	0/5	.	.	0/5	.	.	0/5	.	.	0/5	.
	Tomato juice	1.18	3/5	1.06–3.32	.	0/5	.	.	0/5	.	.	0/5	.	.	0/5	.	13.51	2/5	23.61–43.92
	Black tea	.	0/3	.	.	0/3	.	.	0/3	.	.	0/3	.	.	0/3	.	.	0/3	.
	Corn silk tea	.	0/3	.	.	0/3	.	.	0/3	.	.	0/3	.	.	0/3	.	.	0/3	.
	Barley tea	.	0/3	.	.	0/3	.	.	0/3	.	.	0/3	.	.	0/3	.	.	0/3	.
	Soybean milk	.	0/3	.	.	0/3	.	.	0/3	.	0.52	2/3	0.50–1.04	.	0/3	.	.	0/3	.
	Total	0.18	3/32	1.06–3.32	.	0/32	.	.	0/32	.	0.05	2/32	0.50–1.04	.	0/32	.	2.78	4/32	6.47–43.92
Vegetables	Tomato	.	0/6	.	.	0/6	.	.	0/6	.	.	0/6	.	.	0/6	.	.	0/6	.
	Onion	.	0/5	.	.	0/5	.	.	0/5	.	.	0/5	.	.	0/5	.	.	0/5	.
	Korean cabbage	.	0/5	.	.	0/5	.	.	0/5	.	.	0/5	.	.	0/5	.	.	0/5	.
	Total	.	0/16	.	.	0/16	.	.	0/16	.	.	0/16	.	.	0/16	.	.	0/16	.
Fruits	Apple	.	0/5	.	0.06	1/5	0.3	.	0/5	.	.	0/5	.	.	0/5	.	1.14	1/5	5.69
	Mandarin	.	0/5	.	.	0/5	.	.	0/5	.	.	0/5	.	.	0/5	.	.	0/5	.
	Grape	.	0/5	.	.	0/5	.	.	0/5	.	.	0/5	.	.	0/5	.	0.78	1/5	3.88
	Watermelon	.	0/5	.	.	0/5	.	.	0/5	.	.	0/5	.	.	0/5	.	.	0/5	.
	Total	.	0/20	.	0.01	1/20	0.3	.	0/20	.	.	0/20	.	.	0/20	.	0.48	2/20	3.88–5.69

^1^ Number of positive samples/number of analyzed samples. ^2^ Not detected (below LOD).

**Table 4:** Estimated daily dietary exposure to *Alternaria* toxins (ng/kg b.w./day)

Scenario	Toxins	Daily Dietary Exposure (ng/kg b.w./day)													
		Cereal Grains		Pulses		Nuts and Seeds		Beverages		Seasoning Foods		Vegetables		Fruits	
		LB	UB	LB	UB	LB	UB	LB	UB	LB	UB	LB	UB	LB	UB
Mean food intake	AOH	0.0255	0.2006	0.0000	0.0075	0.0041	0.0053	0.0168	0.0323	0.0362	0.0382	0.0000	0.0353	0.0000	0.0347
	AME	0.0109	0.1061	0.0000	0.0041	0.0430	0.0445	0.0000	0.0446	0.0087	0.0111	0.0000	0.0245	0.0081	0.0297
	ALT	0.0000	0.0924	0.0000	0.0039	0.0008	0.0010	0.0000	0.0243	0.0000	0.0039	0.0000	0.0652	0.0000	0.0642
	ATX-I	0.0078	0.0576	0.0000	0.0021	0.0000	0.0007	0.0079	0.0156	0.0000	0.0019	0.0000	0.0951	0.0000	0.0936
	TEN	0.0267	0.3062	0.0000	0.0119	0.0008	0.0054	0.0000	0.1176	0.0000	0.0141	0.0000	0.3859	0.0000	0.3796
	TeA	2.2538	2.2865	0.0010	0.0076	0.2043	0.2057	0.4041	0.5017	22.9266	22.9266	0.0000	0.4538	0.1759	0.5676
Extreme food intake (P95)	AOH	0.4782	0.9302	0.0000	0.0309	0.0065	0.0111	0.0168	0.0323	0.1527	0.1609	0.0000	0.1675	0.0000	0.2455
	AME	0.2580	0.5018	0.0000	0.0169	0.1414	0.1470	0.0000	0.0446	0.0434	0.0552	0.0000	0.1159	0.0705	0.2192
	ALT	0.0000	0.2507	0.0000	0.0162	0.0022	0.0029	0.0000	0.0243	0.0000	0.0173	0.0000	0.3092	0.0000	0.4532
	ATX-I	0.0428	0.1759	0.0000	0.0088	0.0000	0.0024	0.0079	0.0156	0.0000	0.0078	0.0000	0.4509	0.0000	0.6609
	TEN	0.4108	1.1425	0.0000	0.0493	0.0026	0.0196	0.0000	0.1176	0.0000	0.0662	0.0000	1.8293	0.0000	2.6813
	TeA	11.5228	11.6342	0.0010	0.0287	0.7277	0.7327	0.4041	0.5017	86.0728	86.0728	0.0000	2.1514	1.3746	4.1193

## References

[1] Pinto V.E.F., Patriarca A. (2017). Alternaria Species and Their Associated Mycotoxins.

[2] Meena M., Samal S. (2019). *Alternaria* host-specific (HSTs) toxins: An overview of chemical characterization, target sites, regulation and their toxic effects. Toxicol. Rep..

[3] Arcella D., Eskola M., Gómez Ruiz J.A., European Food Safety Authority (2016). Dietary exposure assessment to *Alternaria* toxins in the European population. EFSA J..

[4] Qiao X., Zhang J., Yang Y., Yin J., Li H., Xing Y., Shao B. (2020). Development of a simple and rapid LC-MS/MS method for the simultaneous quantification of five *Alternaria* mycotoxins in human urine. J. Chromatogr. B.

[5] Pfeiffer E., Eschbach S., Metzler M. (2007). Alternaria toxins: DNA strand-breaking activity in mammalian cells in vitro. Mycotoxin Res..

[6] Schwarz C., Kreutzer M., Marko D. (2012). Minor contribution of alternariol, alternariol monomethyl ether and tenuazonic acid to the genotoxic properties of extracts from *Alternaria alternata* infested rice. Toxicol. Lett..

[7] Solhaug A., Wisbech C., Christoffersen T.E., Hult L.O., Lea T., Eriksen G.S., Holme J.A. (2015). The mycotoxin alternariol induces DNA damage and modify macrophage phenotype and inflammatory responses. Toxicol. Lett..

[8] Aichinger G., Del Favero G., Warth B., Marko D. (2021). *Alternaria* toxins—Still emerging?. Compr. Rev. Food Sci. Food Saf..

[9] Schrader T.J., Cherry W., Soper K., Langlois I., Vijay H.M. (2001). Examination of *Alternaria alternata* mutagenicity and effects of nitrosylation using the Ames Salmonella test. Teratog. Carcinog. Mutagen..

[10] EFSA Panel on Contaminants in the Food Chain (CONTAM) (2011). Scientific opinion on the risks for animal and public health related to the presence of *Alternaria* toxins in feed and food. EFSA J..

[11] Chen A., Mao X., Sun Q., Wei Z., Li J., You Y., Zhao J., Jiang G., Wu Y., Wang L. (2021). *Alternaria* mycotoxins: An overview of toxicity, metabolism, and analysis in food. J. Agric. Food Chem..

[12] Scott P.M., Weber D., Kanhere S.R. (1997). Gas chromatography-mass spectrometry of *Alternaria* mycotoxins. J. Chromatogr. A.

[13] Krska R., Schubert-Ullrich P., Molinelli A., Sulyok M., MacDonald S., Crews C. (2008). Mycotoxin analysis: An update. Food Addit. Contam..

[14] Woo S.Y., Ryu S.Y., Tian F., Lee S.Y., Park S.B., Chun H.S. (2019). Simultaneous determination of twenty mycotoxins in the Korean soybean paste doenjang by LC-MS/MS with immunoaffinity cleanup. Toxins.

[15] Noser J., Schneider P., Rother M., Schmutz H. (2011). Determination of six *Alternaria* toxins with UPLC-MS/MS and their occurrence in tomatoes and tomato products from the Swiss market. Mycotoxin Res..

[16] Zhao K., Shao B., Yang D., Li F., Zhu J. (2015). Natural occurrence of *Alternaria* toxins in wheat-based products and their dietary exposure in China. PLoS ONE.

[17] Xu W., Han X., Li F., Zhang L. (2016). Natural occurrence of *Alternaria* toxins in the 2015 wheat from Anhui province, China. Toxins.

[18] Bansal M., Saifi I.J., Dev I., Sonkar A.K., Dixit S., Singh S.P., Ansari K.M. (2021). Occurrence of Alternariol and Alternariolmonomethyl ether in edible oils: Their thermal stability and intake assessment in state of Uttar Pradesh, India. J. Food Sci..

[19] Gonçalves C., Tölgyesi Á., Bouten K., Robouch P., Emons H., Stroka J. (2022). Determination of *Alternaria* Toxins in Tomato, Wheat, and Sunflower Seeds by SPE and LC-MS/MS—A Method Validation Through a Collaborative Trial. J. AOAC Int..

[20] Zwickel T., Klaffke H., Richards K., Rychlik M. (2016). Development of a high performance liquid chromatography tandem mass spectrometry based analysis for the simultaneous quantification of various *Alternaria* toxins in wine, vegetable juices and fruit juices. J. Chromatogr. A.

[21] Xing J., Zhang Z., Zheng R., Xu X., Mao L., Lu J., Shen J., Dai X., Yang Z. (2021). Simultaneous Detection of Seven *Alternaria* Toxins in Mixed Fruit Puree by Ultra-High-Performance Liquid Chromatography-Tandem Mass Spectrometry Coupled with a Modified QuEChERS. Toxins.

[22] Ji X., Xiao Y., Jin C., Wang W., Lyu W., Tang B., Yang H. (2022). *Alternaria* mycotoxins in food commodities marketed through e-commerce stores in China: Occurrence and risk assessment. Food Control.

[23] Lattanzio V.M., Verdini E., Sdogati S., Bibi R., Ciasca B., Pecorelli I. (2022). Monitoring *Alternaria* toxins in Italian food to support upcoming regulation. Food Addit. Contam. Part B.

[24] Janić Hajnal E., Kos J., Pezo L., Radić B., Malachová A., Krska R., Sulyok M. (2021). Presence of *Alternaria* toxins in maize from Republic of Serbia during 2016–2017. J. Food Process. Preserv..

[25] AOAC International (2016). Appendix F: Guidelines for Standard Method Performance Requirements. https://www.aoac.org/wp-content/uploads/2019/08/app_f.pdf.

[26] Fan Y., Liu F., He W., Qin Q., Hu D., Wu A., Jiang W., Wang C. (2022). Screening of multi-mycotoxins in fruits by ultra-performance liquid chromatography coupled to ion mobility quadrupole time-of-flight mass spectrometry. Food Chem..

[27] Zhao X., Liu D., Yang X., Zhang L., Yang M. (2022). Detection of seven *Alternaria* toxins in edible and medicinal herbs using ultra-high performance liquid chromatography-tandem mass spectrometry. Food Chem. X.

[28] Gambacorta L., El Darra N., Fakhoury R., Logrieco A.F., Solfrizzo M. (2019). Incidence and levels of *Alternaria* mycotoxins in spices and herbs produced worldwide and commercialized in Lebanon. Food Control.

[29] Mujahid C., Savoy M., Baslé Q., Woo P.M., Ee E.C.Y., Mottier P., Bessaire T. (2020). Levels of *Alternaria* toxins in selected food commodities including green coffee. Toxins.

[30] López P., Venema D., de Rijk T., de Kok A., Scholten J.M., Mol H.G., de Nijs M. (2016). Occurrence of *Alternaria* toxins in food products in The Netherlands. Food Control.

[31] Bertuzzi T., Rastelli S., Pietri A., Giorni P. (2021). *Alternaria* toxins in tomato products in Northern Italy in the period 2017–2019. Food Addit. Contam. Part B.

[32] European Committee for Standardization (CEN) Foodstuffs-Determination of *Alternaria* Toxins in Tomato, Wheat and Sunflower Seeds by SPE Clean-up and HPLC-MS/MS (SIST EN 17521:2021), 2021. ITEH STANDARDS. https://standards.iteh.ai/catalog/standards/cen/5b4c4bff-8ed1-40b6-8cde-193db3ca1158/en-17521-2021.

[33] Korean National Health and Nutrition Examination Survey. https://www.khidi.or.kr/nutristat.

